# *In vitro* susceptibility to quinine and microsatellite variations of the *Plasmodium falciparum* Na^+^/H^+^ exchanger transporter (*Pfnhe-1)* gene in 393 isolates from Dakar, Senegal

**DOI:** 10.1186/1475-2875-12-189

**Published:** 2013-06-07

**Authors:** Aurélie Pascual, Bécaye Fall, Nathalie Wurtz, Mansour Fall, Cheikhou Camara, Aminata Nakoulima, Eric Baret, Bakary Diatta, Khadidiatou Ba Fall, Pape Saliou Mbaye, Yaya Diémé, Raymond Bercion, Hervé Bogreau, Sébastien Briolant, Christophe Rogier, Boubacar Wade, Bruno Pradines

**Affiliations:** 1 Département d’Infectiologie de Terrain, Unité de Parasitologie, Institut de Recherche Biomédicale des Armées, Marseille, France; 2 Unité de Recherche sur les Maladies Infectieuses et Tropicales Emergentes, UM 63, CNRS 7278, IRD 198, Inserm 1095, Aix Marseille Université, Marseille, France; 3 Centre National de référence du Paludisme, Marseille, France; 4 Laboratoire d’étude de la chimiosensibilité du paludisme, Fédération des laboratoires, Hôpital Principal de Dakar, Dakar, Sénégal; 5 Service de réanimation médicale, Hôpital Principal de Dakar, Dakar, Sénégal; 6 Service des urgences, Hôpital Principal de Dakar, Dakar, Sénégal; 7 Service de pédiatrie, Hôpital Principal de Dakar, Dakar, Sénégal; 8 Service de pathologie infectieuse, Hôpital Principal de Dakar, Dakar, Sénégal; 9 Département de médecine interne et spécialités médicales de pathologie tropicale, Hôpital Principal de Dakar, Dakar, Sénégal; 10 Laboratoire de Biologie, Institut Pasteur de Dakar, Dakar, Sénégal; 11 Direction Interarmées du Service de Santé, Cayenne, Guyane, France; 12 Laboratoire de Parasitologie, Institut Pasteur de la Guyane, Cayenne, Guyane, France; 13 Institut Pasteur de Madagascar, Antananarivo, Madagascar; 14 Chefferie, Hôpital Principal de Dakar, Dakar, Sénégal

**Keywords:** Malaria, Plasmodium falciparum, Anti-malarial, In vitro, Resistance, Molecular marker, Pfnhe-1, Senegal

## Abstract

**Background:**

Although the World Health Organization recommends replacing quinine (QN) by artesunate due to its increased efficacy and the higher tolerance to the drug in both adults and children, QN remains a first-line treatment for severe malaria, especially in Africa. Investigations of microsatellite *Pfnhe-1* ms4760 polymorphisms in culture-adapted isolates from around the world have revealed that an increase in the number of DNNND amino acid motifs was associated with decreased QN susceptibility, whereas an increase in the number of DDNHNDNHNND motifs was associated with increased QN susceptibility.

**Methods:**

In this context, to further analyse associations between *Pfnhe-1* ms4760 polymorphisms and QN susceptibility, 393 isolates freshly collected between October 2009 and January 2010 and July 2010 and February 2011, respectively, at the Hôpital Principal de Dakar, Senegal were assessed *ex vivo* for QN susceptibility, and their genes were amplified and sequenced.

**Results:**

Of the 393 *Plasmodium falciparum* clinical isolates collected, 145 were successfully cultured. The 145 QN IC_50_s ranged from 2.1 to 1291 nM, and 17 isolates (11.7%) exceed the QN reduced susceptibility threshold of 611 nM. Among the 393 *P. falciparum* clinical isolates, 47 different alleles were observed. The three most prevalent profiles were ms4760-1 (no = 72; 18.3%), ms4760-3 (no = 65; 16.5%) and ms4760-7 (no = 40; 10.2%). There were no significant associations observed between QN IC_50_ values and i) the number of repeats of DNNND in block II (p = 0.0955, Kruskal-Wallis test); ii) the number of repeats of DDNHNDNHNND in block V (p = 0.1455, Kruskal-Wallis test); or iii) ms4760 profiles (p = 0.1809, Kruskal-Wallis test).

**Conclusions:**

*Pfnhe-1* ms4760 was highly diverse in parasite isolates from Dakar (47 different profiles). Three profiles (ms4760-1, ms4760-3 and ms4760-7) were predominant. The number of repeats for block II (DNNND) or block V (DDNHNDNHNND) was not significantly associated with QN susceptibility. New studies, and especially *in vivo* studies, are necessary to confirm the role of *Pfnhe-1* ms4760 as a marker of QN resistance.

## Background

Although the World Health Organization (WHO) recommends replacing quinine (QN) with artesunate due to its improved efficacy and the higher tolerance of the drug in both adults and children [1,2], QN remains a first-line treatment for severe malaria, especially in Africa, and is still used as a second-line therapy in combination with doxycycline, tetracycline or clindamycin for uncomplicated malaria in many countries [3]. Despite the efficacy of QN against chloroquine-resistant *Plasmodium falciparum* isolates, reports of QN resistance (QNR) have been increasing. In the 1980s, the frequency of clinical failures increased in Southeast Asia [4-6], South America [7] and Africa [8,9]. Despite the longevity of QN use, the mechanisms of resistance (and its mode of action) have not yet been resolved. QN, a quinoline derivative, is a monoprotic weak base that accumulates within the low pH environment of the parasite digestive vacuole of *P. falciparum*. QN presumably acts by interference with the detoxification of haem produced during haemoglobin degradation by *P. falciparum* asexual blood stages [10]. The mechanism of QNR is complex and multigenic. QNR has been associated with mutations in both the *P. falciparum* multidrug resistance gene *mdr1* (*Pfmdr1*) [11] and the chloroquine resistance transporter gene *Pfcrt*[12]. More recently, other genetic polymorphisms, such as mutations in the resistance protein gene *Pfmrp*[13], have been suggested to contribute to QNR. PfMRP knockout parasites displayed an increased susceptibility to QN [14]. Using quantitative trait loci (QTL) on the genetic cross of HB3 and Dd2 strains, Ferdig *et al.* identified genes associated with reduced QN susceptibility on chromosome 5, encoding *Pfmdr1*, on chromosome 7, encoding *Pfcrt*, and on chromosome 13, encoding the sodium/hydrogen exchanger gene *Pfnhe-1*[15]. Sequences of *Pfnhe-1* showed multiple and complex variations including point polymorphisms at three separate codons (790, 894 and 950) and microsatellite variations in three different repeat sequences (msR1, ms3580 and ms4760). However, the three point polymorphisms and microsatellite polymorphisms msR1 and ms3580 showed no significant associations with QN susceptibility. Conflicting data have been reported on *Pfnhe-1* polymorphisms. However, the investigations of the microsatellite ms4760 polymorphisms in culture-adapted isolates from around the world showed an association with the QN susceptibility phenotype [16]. A repetition of the amino acid motif DNNND was associated with a decreased susceptibility to QN based on the clinical failure of QN in a traveller from Senegal [17], and data from fresh isolates from Vietnam (n = 79) [18] and from culture-adapted isolates from the China-Myanmar border area (n = 60) [19], Asia, South America and Africa (n = 95) [20]. In 29 cultured-adapted isolates from the Kenya [21] and in 172 freshly obtained isolates from Uganda [22], the duplication of the DNNND motif was associated with a reduced susceptibility to QN compared to isolates with one or more than two repeats. Moreover, an increased number of DDNHNDNHNND motifs were associated with an increased susceptibility to QN [15,16,18-20]. Paradoxically, increased numbers of this latter amino acid motif were associated with a reduced susceptibility to QN based on 83 freshly obtained isolates from Madagascar and 13 African countries [23]. Moreover, these samples did not exhibit any associations between the number of DNNND repeats and QN susceptibility. Furthermore, there was no association between the number of DNNND and DDNHNDNHNND repeats and QN susceptibility based on freshly obtained isolates from the Republic of Congo (n = 74) [24], Thailand (n = 85) [25], Asia, South America and Africa(n = 90) [20].

In this context, to further analyse associations between polymorphisms in *Pfnhe-1* ms4760 and QN susceptibility, 393 freshly obtained isolates from Dakar, Senegal were assessed *ex vivo* for QN susceptibility and their genes were amplified and sequenced.

## Methods

### Reference culture-adapted strains and clinical isolates of *Plasmodium falciparum*

Between October 2009 and January 2010 and July 2010 and February 2011, 393 *P. falciparum* clinical isolates were collected from patients with malaria recruited at the Hôpital Principal de Dakar, a military hospital, in the context of an evaluation of *ex vivo* malaria susceptibility to anti-malarial drugs in Dakar [26,27]. Venous blood samples were collected in Vacutainer® ACD tubes (Becton Dickinson, Rutherford, NJ, USA) prior to patient treatment. Informed verbal consent was obtained from patients and/or their parents before blood collection. An assessment of *P. falciparum* susceptibility to anti-malarial drugs was performed using the same venous blood sample used for this diagnostic. The study was reviewed and approved by the ethical committee of the Hôpital Principal de Dakar.

Thin blood smears were stained using a RAL® kit (Réactifs RAL, Paris, France) and were examined to determine *P. falciparum* density and confirm monoinfection. Parasitized erythrocytes were washed three times with RPMI 1640 medium (Invitrogen, Paisley, UK) buffered with 25 mM HEPES and 25 mM NaHCO_3_. If parasitaemia exceeded 0.5%, infected erythrocytes were diluted to 0.5% with uninfected erythrocytes (human blood type A+) and re-suspended in RPMI 1640 medium supplemented with 10% human serum (Abcys S.A. Paris, France), for a final haematocrit of 1.5%.

### Drugs

QN was purchased from Sigma (Saint Louis, MO, USA) and was dissolved first in methanol and then diluted in water to final concentrations ranging from 5 nM to 3200 nM. Batches of plates were tested and validated using the CQ-susceptible 3D7 strain (West-Africa) and the CQ-resistant W2 strain (Indochina) (MR4, Virginia, USA) in three to six independent experiments using the conditions described in the paragraph below. The two strains were synchronized twice with sorbitol before use [28], and clonality was verified every 15 days using PCR genotyping of the polymorphic genetic markers *msp1* and *msp2* and using microsatellite loci [29,30] and additionally verified each year by an independent laboratory from the Worldwide Anti-malarial Resistance Network (WWARN).

### *Ex vivo* assay

For *in vitro* isotopic microtests, 200 μl of synchronous parasitized red blood cells (final parasitaemia, 0.5%; final haematocrit, 1.5%) was aliquoted into 96-well plates pre-dosed with anti-malarial drugs. The plates were incubated in a sealed bag for 42 h at 37°C with the atmospheric generators for capnophilic bacteria Genbag CO2® at 5% CO_2_ and 15% O_2_ (BioMérieux; Marcy l’Etoile, France) [31]. After thawing the plates, haemolysed cultures were homogenized by vortexing the plates. Both the success of the drug susceptibility assay and the appropriate volume of haemolysed culture to use for each assay were determined for each clinical isolate during a preliminary pLDH ELISA. Both pre-test and subsequent experimental ELISAs were performed using a commercial kit (ELISA-Malaria antigen test, ref 750101, DiaMed AG, Cressier s/Morat, Switzerland) as previously described [32]. The optical density (OD) of each sample was measured with a spectrophotometer (Multiskan EX, Thermo Scientific, Vantaa, Finland).

The concentration at which the drugs were able to inhibit 50% of parasite growth (IC_50_) was calculated with the inhibitory sigmoid Emax model with an estimation of the IC_50_ through non-linear regression using a standard function of the R software (ICEstimator version 1.2) [33]. IC_50_ values were validated only if the OD ratio (OD at concentration 0 / OD at concentration max) was superior to 1.8 and the confidence interval ratio (upper 95% confidence interval of the IC_50_ estimation/lower 95% confidence interval of the IC_50_ estimation) was inferior to 2.0 [33].

### Genotyping of the *Pfnhe* ms4760 microsatellite polymorphisms

Parasite DNA from 100 μl of infected blood was extracted using the E.Z.N.A. Blood DNA kit (Omega Bio-Tek, GA, USA). A sequence containing the previously described ms4760 microsatellite [15] was amplified using *pfnhe*-3802 F 5′-TTATTAAATGAATATAAAGA-3′ and *pfnhe*-4322R 5′-TTTTTTATCATTACTAAAGA-3′ primers. Sequencing was performed using ABI Prism Big Dye Terminator v1.1 Cycle Sequencing Ready Reaction Kits (Applied Biosystems, CA, USA), according to the manufacturer’s instructions. Sequences were analysed with BioEdit sequence alignment editor (version 7.0.9.0) software.

### Statistical analysis

Data were analysed using R software (version 2.10.1). Differences between the QN IC_50_ values of isolates harbouring DNNND repeats, DDNHNDNHNND repeats or profiles were compared using the Kruskal-Wallis test.

## Results

Of the 393 *P. falciparum* clinical isolates collected at the Hôpital Principal de Dakar, 145 isolates were successfully cultured. The 145 QN IC_50_s ranged from 2.1 to 1291 nM, and 17 isolates (11.7%) exceeded the QN-reduced-susceptibility threshold of 611 nM that has been previously defined [31].

**Figure 1:**
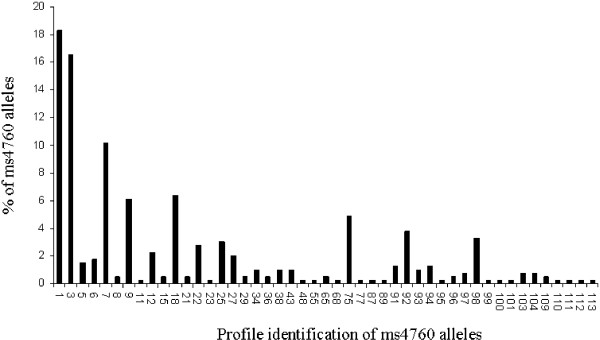
**The distribution of *Pfnhe-1*** **ms4760 profiles among the 393 *P. falciparum *isolates from Dakar, Senegal collected from October 2009 until February 2011.**

**Figure 2:**
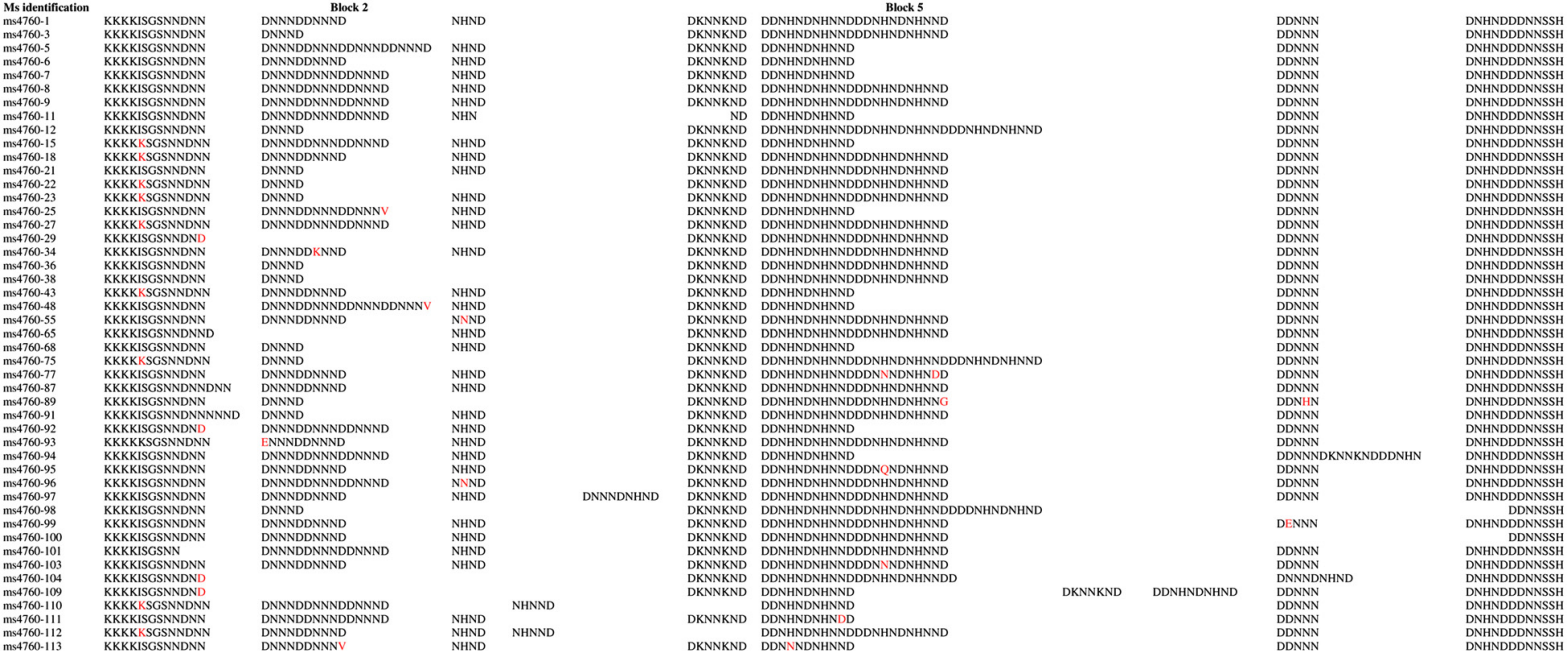
**The amino acid alignment of the 47 *Pfnhe-1*** **ms4760 haplotypes found in the 393 *P. falciparum *isolates from Dakar, Senegal collected from October 2009 until February 2011.**

Among the 393 *P. falciparum* clinical isolates, 47 different alleles were observed, including five profiles not previously described (from ms4760-109 to ms4760-113) (Figure 1). The amino acid sequence alignments of these 47 profiles are described in Figure 2. The three most prevalent profiles were ms4760-1 (no = 72; 18.3%), ms4760-3 (no = 65; 16.5%) and ms4760-7 (no = 40; 10.2%).

**Figure 3:**
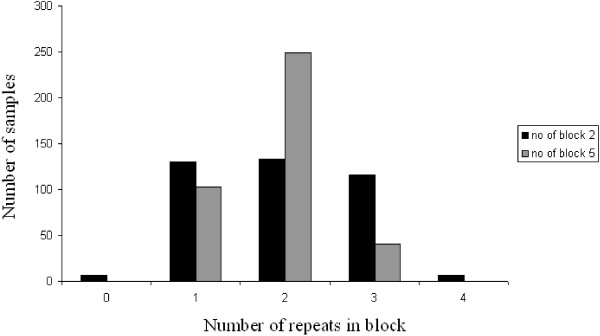
**The distribution of *Pfnhe-1 *profiles according to the number of repeats in block II (DNNND) and block V (DDNHNDNHNND).**

The number of repeats for block II (DNNND) ranged from zero to four, and the number of repeats for block V (DDNHNDNHNND) ranged from one to three (Figure 3). For block II, groups with one repeat (33.1%), two repeats (33.8%) and three repeats (29.5%) represented the majority of the 393 isolates. For block V, the group with two repeats alone represented 63.4% of the isolates.

There was no observed significant association between QN IC_50_ values and the number of repeats of DNNND in block II (p = 0.0955, Kruskal-Wallis test) (Table 1). The 145 isolates were classified into two groups: < 2 repeats of DNNND and ≥ 2 repeats. The QN IC_50_ values were not significantly different in the group < 2 repeats of DNNND in block II (mean IC_50_ = 184.1 nM, 95% confidence interval 134.6-252.4) compared with the group ≥ 2 repeats of DNNND in block II (mean IC_50_ = 153.5 nM, 95% confidence interval 123.3-191.0) (p = 0.2224, Kruskal-Wallis test).

There was no observed significant association between QN IC_50_ values and the number of repeats of DDNHNDNHNND in block V (p = 0.1455, Kruskal-Wallis test) (Table 2). The 145 isolates were classified into two groups: < 2 repeats of DDNHNDNHNND and ≥ 2 repeats. The QN IC_50_ values were not significantly different in the group < 2 repeats of DDNHNDNHNND in block V (mean IC_50_ = 134.0 nM,

 95% confidence interval 95.9-187.1) in comparison to the group ≥ 2 repeats of DDNHNDNHNND in block V (mean IC_50_ = 175.8 nM, 95% confidence interval 142.2-217.3) (p = 0.1084, Kruskal-Wallis test).

Additionally, there was no observed significant association between QN IC_50_ values and ms4760 profiles (p = 0.1809, Kruskal-Wallis test) (Table 3).

## Discussion

QN has been used to treat malaria for more than 350 years in Africa, with little emergence and spread of resistance. Although WHO recommends replacing QN with artesunate, QN remains the first-line anti-malarial treatment for complicated malaria in Europe and Africa. Although QN has retained good anti-malarial efficacy in most areas, its clinical efficacy has decreased in some regions. The first cases of QN clinical failure were observed in Brazil and Asia in the 1960s; then in the 1980s, clinical failures became more frequent in Southeast Asia, South America and Africa [4-9]. However, QN resistance is not yet a significant problem in Africa, and QN remains both the first-line drug used to treat severe malaria and a second-line therapy for uncomplicated malaria in some areas of Africa.

Even in areas where QN remains effective, such as sub-Saharan Africa, the susceptibility of individual *P. falciparum* isolates to QN has varied widely. The IC_50_s for isolates collected in Senegal were 31 to 765

 nM in 1984 (Thies and Kaolack) [34], 5 to 932 nM in 1996 (Dielmo) [35] and 6 to 1291 nM in 2009 (Dakar) [26]. Many other studies have reported wide ranges of susceptibility to QN: 25 to 1253 nM in Comoros [36], 36 to 1097 nM in the Republic of Congo [24] (studies assessed in the same conditions as studies in Senegal in 1996 and 2009) or 15 to 761 nM in Uganda [22]. The wide range of QN susceptibility and recent evidence for QN treatment failure seen across Africa [8,9,17] suggest that the evolution of parasites with reduced susceptibility may contribute to decreased QN efficacy.

Although some reports of QN treatment failure exist, it is difficult to confirm QN resistance because of the drug’s short elimination half-life, the requirement to administer it three times a day for at least five days, drug intolerance that often leads to poor compliance and a lack of reliable data on the correlation between QN IC_50_ and clinical failure.

Even if WHO recommends replacing QN with artesunate as first-line anti-malarial treatment for complicated malaria, maximizing the efficacy and longevity of QN remains important and will depend critically on the pursuit of intensive research towards the identification of *in vitro* markers of QNR and the implementation of *ex vivo* and

**Table 1:** **The distribution of QN IC**_**50 **_**according to the number of repeats in block II (DNNND) of *Pfnhe-1*** **ms4760**

**Number of repeats**	**Number of samples**	**Geometric mean (nM)**	**95% Confidence interval**
0	1	81.1	
1	51	187.1	136.1-257.6
2	48	189.7	141.6-254.1
3	41	126.8	89.1-179.9
4	4	88.1	24.5-316.9

*in vivo* surveillance programs, such as those championed by the WorldWide Antimalarial Resistance Network [37,38]. Specifically, there is a need to identify molecular markers that effectively predict QN resistance and enable the active surveillance of temporal trends in parasite susceptibility [39]. The present study aimed to evaluate the association between the *Pfnhe* polymorphism and QN susceptibility in freshly obtained isolates from Dakar, Senegal to assess the validity of *Pfnhe* as a molecular marker of QN susceptibility in this region.

Prior to this report, no data were yet available on the sequence variation of *Pfnhe-1* ms4760 in *P. falciparum* parasites from Senegal. Among the 393 studied sequences, 47 different alleles were observed in samples from Dakar. Consistent with previous reports [22,24,40], *Pfnhe-1* ms4760 was highly diverse among parasite isolates. It appears that polymorphisms are more important in Africa and the Indian Ocean region than in India or Asia: in Senegal, 47 different profiles (393 samples) were observed; in the Republic of Congo, 27 different profiles (74 samples) [24]; in Uganda, 40 different profiles (172 samples) [22]; and in the Indian Ocean, 29 different profiles (595 samples) [40], whereas in Vietnam, only ten different profiles (79 samples) were observed [18]; in the China-Myanmar border area, ten different profiles (60 samples) [19]; and

**Table 2:** **The distribution of QN IC**_**50 **_**according to the number of repeats in block IV (DDNHNDNHNND) of *Pfnhe-1*** **ms4760**

**Number of repeats**	**Number of samples**	**Geometric mean (nM)**	**95% Confidence interval**
1	37	129.4	92.7-181.1
2	94	168.7	133.7-213.3
3	14	228.6	138.7-376.7

**Table 3:** **The distribution of QN IC**_**50 **_**according to the major profiles of *Pfnhe-1*** **ms4760**

**Profiles**	**Number of samples**	**Geometric mean (nM)**	**95% Confidence interval**
Ms4760-1	25	234.4	166.3-330.4
Ms4760-3	27	181.6	109.1-302.0
Ms4760-7	14	123.0	77.5-195.9
Ms4760-18	11	212.3	122.2-369.0
Ms4760-other	68	140.3	106.4-185.4

 in India, 16 different profiles (244 samples) [41]. This situation likely reflects the level of transmission in these areas and the level of QN selection pressure. The genetic diversity of ms4760, assessed by Nei’s unbiased expected heterozygosity (He), was significantly higher in African isolates (ranged from 0.66 to 0.85) and Indian isolates (0.68) than in Asian isolates (0.49 to 0.68) [40]. Only three profiles (ms4760-1, ms4760-3 and ms4760-7) of the four expected predominant profiles (ms4760-1, ms4760-3, ms4760-6 and ms4760-7) [18-24,40,41] were found to predominate in Senegal. Profile ms4760-6 represents only 1.8% of the studied sequences. The predominance of these three profiles combined with a low rate of the ms4760-6 profile was also found in parasites collected before QN treatment and in recurrent parasites in Mali [42].

The 145 QN IC_50_s ranged from 2.1 to 1291 nM and, in 17 isolates (11.7%), exceeded the QN-reduced-susceptibility threshold of 611 nM that has been previously defined [31]. In this study, the number of repeats for block II (DNNND) or block V (DDNHNDNHNND) was not significantly associated with QN susceptibility. These data are similar to those found in freshly obtained isolates from Asia, South America and Africa [20], the Republic of Congo [24] or Thailand [25]. The reduced susceptibility to QN was associated with an increased number of DNNND repeats or with two repeats, and the increased susceptibility to QN associated with an increased number of DDNHNDNHNND repeats was found more often in culture-adapted parasites [19-21]. Given that the influence of *Pfnhe* on QN susceptibility has been shown to be parasite-dependent, these apparently conflicting results may be explained, in part, by differences in the geographic origin of the parasites analysed, as their local selection history and genetic background varies, and by the method used to assess *in vitro* susceptibility to QN (i.e. an *in vitro* test for culture-adapted isolates or strains versus an *ex vivo* test for freshly obtained isolates) [20].

One explanation for these differences could be variation in genetic background. A specific genetic background observed in Asia may allow the observed contribution of *Pfnhe* polymorphism to QN *in vitro* susceptibility. This explanation is consistent with the following: i) the first evidence of *Pfnhe*-QN resistance association from QTL analysis using Americano-Asian cross strains [15], ii) that most associations identified have been shown among Asian strains [15,16,18,19] and iii) that at least five genes spanning the *P. falciparum* genome influence the QNR *in vitro* phenotype with an additive effect or with pairwise interactions [15].

Additionally, these genetic dissimilarities between African and Asian *Plasmodium* populations may be accentuated by different local selection histories. The best-documented genomic modifications by a local selection process relate to drug pressure. Several studies have shown that drug pressure may involve extended linkage disequilibrium around a drug resistance associated gene [43]. This is characterized by a strong loss of genetic diversity called a selective sweep. This process stretch may be modified by either i) drug use or ii) malaria transmission level. A reduced drug use and higher malaria transmission level in Africa would be consistent with the lower selective sweep and an absence of linkage disequilibrium between *Pfnhe* and other cooperative drug response genes or selected compensatory mutations. For example, the QTL analysis of chromosome 13 located 60 genes of unknown function as being close to *Pfnhe*[15]. They would be more or less linked depending on drug pressure and malaria transmission levels if QN pressure selects at least one of them. In Kenya, there is an association between two DNNND repeats in the ms4760 *Pfnhe* microsatellite and a reduced susceptibility to QN in 29 *P. falciparum* isolates [44]. This is consistent with the historic precedent of the spread of drug resistance around the world. The emergence of chloroquine resistance in Asia was followed by an initial introduction into East Africa and subsequent spread across the African continent. Geographic proximity may explain plasmodial population migration. Moreover, East African plasmodial populations may exhibit genetic dissimilarities to other African populations [29].

As the QN response is controlled by multiple genes with complex interactions, one would expect: i) a higher sensitivity to genetic background than if the response were controlled by only one gene, ii) higher sensitivity to parameters that might cause linkage disequilibrium between genes and iii) various combinations of gene polymorphisms that might result in similar QN resistance phenotypes.

The three profiles (ms4760-1, ms4760-3 and ms4760-7) were predominant in parasites collected before QN treatment and in recurrent parasites in Mali [42]. The prevalence of ms4160-1 increased significantly from 26.2% to 46.3% after QN treatment in recurrent parasites. QN treatment selected for the *Pfnhe-1* ms4760-1 profile. The profiles ms4760-3 and ms4760-18 were found in recurrent parasites from a patient returning from French Guiana and Senegal who failed with QN treatment [17,45]. Such *in vivo* studies are necessary to confirm the role of *Pfnhe-1* ms4760 as a marker of QN resistance. However, according to both previous studies and this current one, as *Pfnhe-1* exhibits inconsistent association with QN susceptibility, the current problem should be to identify the new other marker that will be best associated with reduced QN susceptibility.

## Conclusion

*Pfnhe-1* ms4760 was highly diverse in parasite isolates in Dakar (47 different profiles). Genetic diversity should be assessed. Three profiles (ms4760-1, ms4760-3 and ms4760-7) were predominant. The number of repeats for block II (DNNND) and block V (DDNHNDNHNND) was not significantly associated with QN susceptibility. New studies, and especially *in vivo* studies, are necessary to confirm the role of *Pfnhe-1* ms4760 as a marker of QN resistance. Studies should aim to identify partners of *Pfnhe-1* or other polymorphisms linked to *Pfnhe-1*.

## Competing interests

The authors declare that they have no competing interests.

## Authors’ contributions

AP and NW carried out the molecular genetic studies. BF, ER and BP carried out the *ex vivo* evaluation of QN susceptibility. MF, CC, AN, BD, KBF, PSM, YD, RB and BW supervised, carried out and coordinated the field collections of patient isolates. BP conceived and coordinated the study. SB, HB, CR and BP analysed the data. NW, AP, SB, HB, BF and BP drafted the manuscript. All the authors read and approved the final manuscript.
